# Knowledge, Attitudes, Practices and Biomonitoring of Farmers and Residents Exposed to Pesticides in Brazil

**DOI:** 10.3390/ijerph9093051

**Published:** 2012-08-24

**Authors:** Juliana Oliveira Pasiani, Priscila Torres, Juciê Roniery Silva, Bruno Zago Diniz, Eloisa Dutra Caldas

**Affiliations:** Laboratory of Toxicology, Department of Pharmaceutical Sciences, Faculty of Health Sciences, University of Brasilia, Campus Universitário Darcy Ribeiro, 709010-9000 Brasília, DF, Brazil; Email: jujuop@gmail.com (J.O.P.); pritfarm@gmail.com (P.T.); jucievasconcelos@gmail.com (J.R.S.); zago.diniz@gmail.com (B.Z.D.)

**Keywords:** KAP study, acetylcholinesterase, butyrylcholinesterase, Brazil

## Abstract

In this study, the knowledge, attitudes and practices regarding pesticide use and the levels of exposure of farmers and residents to organophosphorous and/or carbamates pesticides were evaluated in two rural settings in Brazil. A questionnaire was completed by 112 farm workers aged ≥18 years. Almost all farmers acknowledged that pesticides were potentially harmful to their health (87.5%); however, over half rarely (48.2%) or never (7.2%) used personal protective devices (PPDs). An association was found (*p* = 0.001) between the work regimen and the use of PPDs, with more frequent equipment use among hired laborers than those involved in family agriculture. A significant correlation (*p* = 0.027) was found between the reporting of adverse symptoms and the use of backpack sprayers. Mean AChE activities of farmers (n = 64) and residents (n = 18) during the exposure and non-exposure periods were significantly lower than their control groups. Mean BChE activities of farmers and residents were significantly lower than their controls during the exposure period. Among the 60 farmers that had blood samples collected in both the exposure and non-exposure (baseline) periods, 10 (16.7%) had AChE depletion of over 30% during the exposure period compared with the baseline level. Six residents living on the same farms also presented this depletion. AChE was over 30% higher than the baseline level for 19 farmers (31.7%), indicating a reboot effect. Special education programs are needed in these regions to promote the safe use of pesticides in the field to decrease the risks from exposure to pesticides for farmers, and from secondary exposure to these compounds for their families.

## 1. Introduction

The use of pesticides is currently the main pest management strategy to guarantee the World’s food supply. Most pesticides, however, are toxic to non-target species, including humans, and the extensive use of these products in the field can lead to occupational diseases and poisonings [1,2,3]. Brazil is one of the largest pesticide users in the World [4], and over 90% of farmers rely on pesticides for pest management [5]. As of July 2012, 46.5% of the 1,541 pesticide products registered in the country were classified as extremely or highly toxic to humans (classes I and II) [6]. The number of pesticide poisoning incidents has increased continuously in the country over the last two decades [7], and the number of occupational accidents related to pesticides has increased by about 40% between 2003 and 2009 [4]. 

Pesticide exposure in the field occurs mainly through dermal contact and inhalation [8], and the use of personal protective devices (PPDs) can help reduce exposure levels or identify early effects before irreparable disease develops [8,9]. Studies aimed at determining the knowledge, attitudes, and practices (KAP) regarding pesticide use have been conducted worldwide to understand the occupational settings and work conditions in which pesticides are handled and applied by farm workers [2,10,11,12,13,14]. 

Organophosphate (OP) and carbamate (CAR) insecticides are used extensively in Brazilian agriculture, mainly in small farm settings [3,14]. They are among the most acute toxic pesticides on the market worldwide, and their registration is being phased out or has been canceled in many countries, including Brazil [15,16,17]. OPs and CARs are inhibitors of acetylcholinesterase (AChE—EC 3.1.1.7), an enzyme responsible for the hydrolysis of the neurotransmitter acetylcholine [18]. A single or repeated exposure to AChE inhibitors leads to the accumulation of acetylcholine in the synaptic cleft, and may cause excessive stimulation of muscarinic and nicotinic receptors throughout the body, producing toxic effects such as nausea, bronchoconstriction, sialorrhea, hypertension and tremor, and affecting the central nervous system [18,19,20]. These insecticides also inhibit plasma butyrylcholinesterase (BChE—EC 3.1.1.8), an enzyme whose physiological function is unknown [18]. The measurement of erythrocyte AChE and plasma BChE represents a reliable way of determining exposure to OPs and CAR, or to monitor occupationally-exposed workers [21,22]. While BChE is considered to be a more sensitive indicator of OP absorption, AChE is a reliable indicator of acute intoxication by anticholinesterase pesticides [23,24].

The aims of this study were to assess the knowledge, attitudes, and practices regarding the use of pesticides by farmers in two rural settings in Midwestern Brazil, and to determine the levels of exposure of farmers and residents to organophosphorous and carbamate pesticides through AChE and BChE analysis. This is the first time that a KAP/biomonitoring study has been conducted in an agricultural setting in this region of the country. 

## 2. Methods

This study was conducted according to international guidelines for the protection of human subjects and was approved by the ethics committee of the University of Brasilia. All participants read and signed the free and informed Consent Term, which included the research objectives, procedures, and privacy in data handling.

### 2.1. Population Studied

This was a cross-sectional epidemiologic study conducted between 2009 and 2011 with farmers from two locations in the Midwestern region of Brazil: Goianápolis (162.38 km^2^, located 170 km from Brasília, the capital of Brazil) and Taquara Rural Nucleus, in the city of Planaltina (351 km^2^, 90 km from Brasília). All participants were 18 years of age or older. All farmers were directly involved with pesticides, either in the preparation of the pesticide solution and/or its application in the field. The study used a convenience sampling (non-probability sampling), through a systematic recruitment process.

In Goianápolis, 40% of its approximately 11,000 inhabitants are directly or indirectly involved in tomato-growing activities [25]. At this location, the crop is cultivated year-round in about 30 planting areas of 1–2 ha, in which 9–10 farmers work in a sharecropper regime under the management of the owner or some other person. After harvesting, the workers migrate to another planting area, repeating the cycle. The managers of 21 planting areas were contacted by telephone (numbers provided by the local seed supplier) to obtain permission to visit the areas, and to recruit farmers to participate in the study. Only two managers agreed to join the study, and we were able to recruit 18 farmers from two planting areas in the region. 

According to EMATER-DF, a local government agency that provides technical support to farmers, there are 318 planting areas and 1,100 farmers in Taquara, mainly vegetable and fruit growers. In each 4–5 ha planting area, 5–13 individuals work in family farming settings or share-cropper regimes growing multiple crops. After harvesting, a new planting cycle begins, usually with a different crop. Our first contact with the farmers took place at two community events sponsored by EMATER, at which 30 farmers agreed to participate in the study, answering a questionnaire and giving us their contact information. We obtained the telephone contact information of an additional 112 farmers from EMATER, 30 of whom were unreachable, 10 were no longer involved in agricultural activities, six did not want to participate in the project, and two were no longer living in the area. A total of 94 farmers from 38 Taquara planting areas participated in the study. In addition, 18 family members not directly involved with pesticides (residents) agreed to participate in the study. For the control group, 64 individuals were recruited from the local hospital, school and EMATER office, in addition to rural workers with no contact with pesticides. An interview was conducted with each potential control group member to assure that none of them had any direct or indirect contact with pesticides over the last year. In summary, the study involved 112 farmers, 18 residents, and 64 controls. 

### 2.2. The Knowledge, Attitudes and Practices (KAP) Study

The farmers participating in the study answered a questionnaire which was applied by a trained professional. The questionnaire, adapted from a previous one developed and validated by our group [26], was comprised of 59 objective questions (yes/no or multiple choice), and one subjective question (concerning adverse symptoms after pesticide exposure). The aim was to obtain information on the workers and the farms, the attitudes and practices of farmers regarding pesticide use, and symptoms following pesticide application to determine the impact of pesticide use on human health and the environment. 

### 2.3. AChE and BChE Activity

The participating farmers, residents and control group were asked to donate blood samples to measure cholinesterase activity. All samples were collected in 4 mL vacuum tubes. In Goianápolis, blood samples were collected by health professionals from the local Secretary of Health. In Taquara, they were collected by a trained pharmacist involved in the project. Samples were sent to the Laboratory of Toxicology within a maximum of 4 hours after collection. 

In this study, a farmer was considered to be in the exposure period up to 5 days after using ChE inhibitor pesticides (organophosphorous and/or carbamates) and in the non-exposure period after 15 days not using ChE inhibitor pesticides. Information about ChE inhibitor pesticide use was obtained directly from the farmers before sample collection. Blood samples from the residents were collected at the same time as those from the farmers. 

Upon arrival at the laboratory, blood samples were centrifuged, the erythrocyte portion hemolysated in a buffer solution (0.02 M, pH 7.6), and the fractions kept frozen (−20 °C) until analyzed. A modified Ellman method [27,28] was used, which is based on the hydrolysis of the substrate (acetylthiocholine or butyrylthiocholine) by each enzyme and reaction of the formed thiol group with ditionitrobenzoic acid (DTNB), yielding nitrobenzoic acid, which was quantified at 420 nm (Shimadzu UV/VIS 1650 PC spectrophotometer). Enzyme activity was determined against a standard curve of L-cysteine (R^2^ = 0.9992), which underwent the same colorimetric reaction. AChE (µmoles/min/mL) and BChE (μmoles/min/mg protein) were determined within 3–5 and 1–3 days after blood collection, respectively, periods in which the enzyme activities did not vary significantly (n = 5, *p* = 0.002). Precision ranged from 3.5 to 14.5% for AchE, and from 1.7 to 4.1% for BChE. Acetylthiocholine, butyrylthiocholine, L-cysteine, albumin and DTNB were purchased from Sigma Aldrich^®^. Folin reagent (for protein determination) was obtained from Merck KGaA^®^, and sodium phosphate tribasic dodecahydrate (for the buffer solution) was obtained from Vetec Quimica Fina.

### 2.4. Data Treatment

The data gathered from the questionnaires and the results of the blood analyses were transferred to the EpiInfo Software 2000 package (Epidemiological Program Office, CDC, Atlanta, GA, USA). The statistical analysis was performed with the IBM SPSS Statistics Version 19 software for Windows. The Fisher test and *X*^2^ were used to determine possible association between the nominal variables of the study. The T-test or Mann-Whitney test was used for the comparison of the means. The level of significance was set at 95% (*p* < 0.05).

## 3. Results

### 3.1. KAP Study

A total of 112 farmers answered the questionnaire; 94 (83.9%) from Taquara, and 18 from Goianápolis. All Goianápolis farmers were tomato growers, and 92% of Taquara farmers were either tomato and/or sweet pepper growers. All except one farmer were male (99.1%). There were no statistical differences in the parameters shown in Table 1 and Table 2 for the individuals of both communities, and thus they were grouped for discussion. 

**Table 1:** Social and demographic characteristics of the Taquara and Goianápolis farmers (N = 112).

Characteristics		n	(%)
Age			
	18–20	8	(7.1)
	21–30	27	(24.1)
	31–40	34	(30.5)
	41–50	25	(22.3)
	51–60	9	(8.0)
	>60	9	(8.0)
Education			
	Illiterate/no schooling	7	(6.3)
	Incomplete primary	55	(49.1)
	Complete primary/incomplete high school	33	(29.4)
	Complete high school/incomplete college	17	(15.2)
Name used to designate pesticides			
	Poison	78	(65.4)
	Agrotoxic	31	(26.1)
	Remedy	4	(3.4)
	Pesticide	2	(1.7)
	Other	4	(3.4)
Years of pesticide use			
	Up to 5	26	(23.2)
	5–10	31	(27.7)
	10–20	31	(27.7)
	20–30	16	(14.3)
	>30	8	(7.1)
Work Regimen			
	Sharecropper or employee	69	(61.6)
	Family agriculture	43	(38.4)
Consumption of alcoholic beverages		63	(56.2)

**Table 2:** Knowledge, attitudes and practices of the Taquara and Goianápolis farmers regarding the use of pesticides (N = 112).

			n	(%)
Hours working in the field				
	More than 8		60	(53.6)
	5–8		48	(42.8)
	Up to 5		4	(3.6)
Storage of pesticide products				
	In a special storage location (outside the house)		87	(77.6)
	With other farm products		19	(17.0)
	In the house		5	(4.5)
	Other		1	(0.9)
Type of pesticide applicator				
	Manual backpack sprayer		45	(40.2)
	Automated static sprayer		41	(36.6)
	Automated backpack sprayer		12	(10.7)
	Open tractor		10	(8.9)
	Other		4	(3.6)
Empty pesticide containers are				
	Turned over to government collection posts		92	(82.1)
	Buried/burned		18	(16.1)
	Did not know		2	(1.8)
Pesticides are necessary in the field			108	(96.4)
Work in the field can impair his/her health			87	(77.7)
Pesticides are harmful to the health of				
	Those who apply the pesticides		91	(81.3)
	Those who work on the farm		79	(70.5)
	Those who consume the crop		68	(60.7)
	Those who live near the planting area		30	(26.8)

Table 1 shows the social and demographic characteristics of the studied populations. The average age of the farmers was 37.7 years (±12.5), most of whom (54.6%) were between 21 and 40 years of age. Almost half of respondents (49.1%) had not completed primary education, and 6.3% were illiterate or had never attended school. The preferred word to designate pesticide products was “poison” (65.4%), followed by *agrotóxico* (26.1%), the legal term used for pesticides in Brazil [29], which may be translated as agrotoxic. Most farmers worked as sharecroppers or employees, and 38.4% worked in a family agricultural setting (all from Taquara). Most of the respondents (76.8%) had been using pesticides for at least 5 years, and 21.4% for over 20 years. Most of the farmers declared being consumers of alcoholic beverages (Table 1).

Table 2 shows the knowledge, attitudes and practices regarding pesticides among the farmers. Most of the respondents (53.6%) work over 8 hours a day. For almost 80%, there was a special room for pesticide storage, and only five individuals (4.5%) reported storing these products inside their homes. At least 40% used a manual backpack sprayer, and 36.6% used an automated static sprayer. The great majority (82.1%) reported turning over the empty pesticide containers to the government container disposal program, and 16.1% buried and/or burned the containers. No significant correlation was found between storage of pesticide products in a reserved/special deposit outside the house and the level of education.

Over 95% of farmers considered pesticides necessary in the field (Table 2), and 77.7% stated that work in the field could impair his/her own health. Almost 90% of the farmers considered pesticides harmful to the health, mainly to those who apply the pesticides or work on the farm (81.3 and 70.5%, respectively); only one-third considered that the health of those living near the plantation area could be impaired by pesticides. A significant correlation (*p* < 0.01) was found among those who thought that his work was harmful to the health, and those who thought that pesticides were harmful to health. Most farmers (67%) agreed that pesticide residues remained in the food after treatment and about 40% of these acknowledged that the amount remaining in the food for consumption depends on the withholding period. For 25.3%, residues in the food remained for a week or less after the last application, 16% for a day or less, 9.3% for a month or less, and 5.3% for over a month. About 60% of farmers considered pesticides harmful to the health of those who eat the treated crop (Table 2).

Most of the farmers purchased the pesticide products at the local cooperative (56.4%), and washed the pesticide application equipment in the field (57.1%). Almost half (42.9%) reapplied the leftover pesticide solution on the same crop and on the same day, 25% disposed it in the soil or in the rivers/brooks, and 23.2% kept it for subsequent application. Only 8.9% of the farmers prepared the exact volume to be used on the crop. Almost all farmers (99.1%) considered the use of PPD necessary during pesticide application, but almost half (48.2%) did not use them properly (Table 3), and 7.2% never used them at all. Gloves were never used by about 18% of the farmers, and impermeable clothes were used by only 18.8% of the farmers. Sharecroppers were more likely to use PPD than farmers in a family agriculture setting (*p* = 0.001). Considering those who used impermeable clothes (always/sometimes), 39.3% had them washed at home (mainly by the wives), 32.6% in the field, 14.6% let them dry and washed them only on weekends, 7.9% washed them either in the field or at home, and 3.4 % never washed them.

**Table 3:** Use of personal protective devices (PPD) by the Taquara and Goianápolis farmers (N = 112).

PPD use	n (%)		
	Complete	Incomplete	No use
	50 (44.6)	54 (48.2)	8 (7.2)
	Always	Sometimes	Never
Boots	88 (78.6)	15 (13.4)	9 (8.0)
Hat	78 (69.6)	19 (17.0)	15 (13.4)
Gloves	64 (57.1)	28 (25.0)	20 (17.9)
Mask	81 (72.3)	18 (16.1)	13 (11.6)
Impermeable clothes	66 (58.9)	25 (22.3)	21 (18.8)
Other	2 (1.8)	-	-

Most of the respondents (55.3%) declared receiving information about pesticides from government extension agents, technicians and/or pesticide sellers, and 19.6% from the cooperatives. The majority of the individuals (65.7%) always followed the orientation received, and 87.5% observed the withholding period. Over half of the farmers (54.5% of 88 respondents) followed the agronomic prescription, but 28.5% did not know what an agronomic prescription was. This prescription is a legal requirement for the purchase of pesticide products in Brazil [29], and is considered a confirmation that a certified technician visited the area and evaluated the crops to which the product is to be applied. Most of the farmers (68.8%) read the product labels with instructions on use, and 58% read the warnings and precautions. Almost all (92.9%) selected the best time for pesticide application (early in the morning and/or at the end of the day), and observed the direction of the wind at the time of application (87.5%). The majority of farmers (62.5%) considered that the information they were provided on pesticides was sufficient to prevent harming their health, 50% to prevent harming other people’s health, and 48.2% to prevent harming the environment. Over 63% answered that pesticides were harmful to animals, and 75.9% that they were harmful to the environment.

Almost one fourth of farmers (26, 23.2%) reported having adverse symptoms after the use of pesticides sometime during their lives. The main symptoms reported were cephalea (13 farmers), dizziness (10 farmers), and vomiting (four farmers). No significant correlation was found between the reporting of adverse symptoms and age, level of education, years of pesticide use, living on the farm, use of PPD, disposal of empty pesticide containers, or hours of work. However, a significant correlation (*p* = 0.027) was found between the reporting of adverse symptoms and the use of backpack sprayers. Among those farmers who reported adverse symptoms, seven (26.9%) reported having been intoxicated by pesticides, from which three were diagnosed by a physician, and two were hospitalized and needed to interrupt their usual activities. Although not significant, the consumption of alcoholic beveranges with some frequency had the second highest correlation with the reporting of some adverse symptom (*p* = 0.052). 

Fifty eighty farmers (51.8% of the participants) answered the question regarding the last OP or CAR pesticide they had applied on the crops. Chlorpyrifos, triazophos and phenthoate were the most reported OPs (31, 27.6 and 19.0%, respectively), in addition to methamidophos (six farmers), acephate (four farmers), and profenophos (one farmer). Carbofuran and methomyl were the only carbamates cited (by two farmers).

### 3.2. AChE and BChE Activities

**Figure 1:**
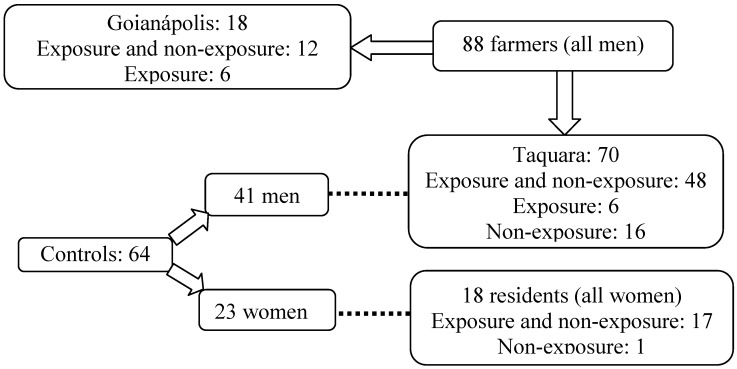
Participation of the individuals in the biomonitoring study.

Of the 112 farmers who participated in the study, 60 who reported using organophosphorous and/or carbamates pesticides agreed to donate blood samples for cholinesterase analysis according to the defined protocol (48 from Taquara and 12 from Goianápolis; exposure and non-exposure period), 16 farmers donated blood only during the non-exposure period, and 12 only during the exposure period (Figure 1). All blood donors were men. Blood samples were taken from 17 residents from Taquara (all women) (41 years ± 14.5, on average) during the farmers’ non-exposure and exposure periods and from one resident during the non-exposure period. The control group was comprised of 64 individuals from Taquara: 41 men (mean age: 35.4 ± 9.2 years) and 23 women (mean age: 32.7 ± 10.6 years). The men in this group were considered the control for the Taquara farmer group and the women the control for the resident group. A diagram showing how the individuals participated in the biomonitoring study is shown in Figure 1.

**Figure 2:**
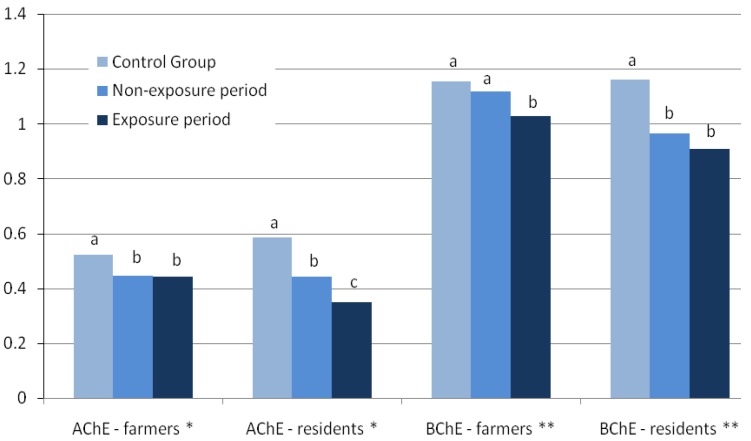
Mean enzyme activities of the Taquara farmers and resident groups during the non-exposure (n = 64 and 18, respectively) and exposure periods (n = 54 and 17, respectively) compared with their respective controls (n = 41 and 23, respectively). * µmol/min/mg of protein; ** µmol/min/mL of plasma. For each enzyme/group, bars with different letters have statistically different means (*p* < 0.05).

Figure 2 shows the mean enzyme activities of the Taquara farmer and resident groups for the non-exposure and exposure periods compared with their respective controls. No significant differences were found between the mean AChE activities of the farmer group during the non-exposure (0.445 µmol/min/mg prot, n = 64) and exposure periods (0.443 µmol/min/mg prot, n = 54); they were, however, statistically lower than the activity found in the men’s control group (0.519 µmol/min/mg prot, n = 41; *p* < 0.02). Mean AChE activity of the women’s control group (0.586 µmol/min/mg prot, n = 23) was significantly higher than that of the resident group for both periods (*p* < 0.01). Additionally, AChE activity in this group was significantly higher during the non-exposure period when compared with the exposure period (0.352 and 0.314 µmol/min/mg prot, respectively) (*p* = 0.011). Mean BChE activity of the farmers during the non-exposure period (1.12 µmol/min/mL plasma) was similar to the control, but higher than that of the exposure period (1.03 µmol/min/mL, *p* = 0.02). The resident group had lower BChE activity (0.964 and 0.910 µmol/min/mL for the non-exposure and exposure periods, respectively) than the control group (1.11 µmol/min/mL) (*p* < 0.05). 

**Figure 3:**
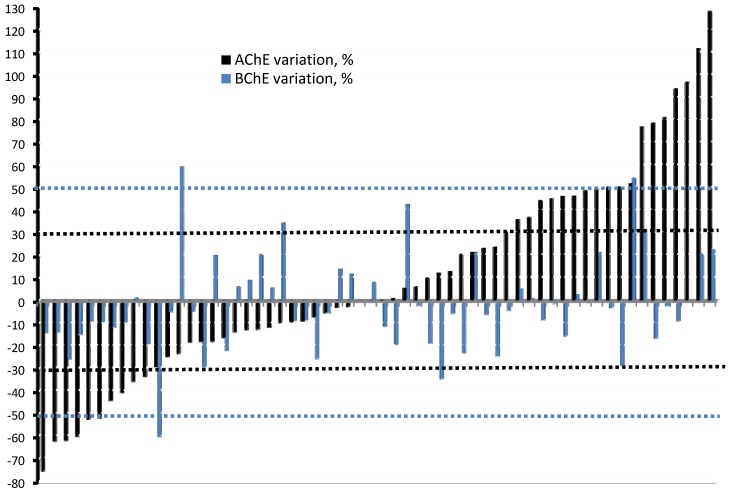
Variation (%) in AChE or BChE activity for each Taquara and Goianápolis farmer (n = 60) between the non-exposure and exposure periods.

Figure 3 shows the variation (%) in AChE and BChE activity for each of the 60 farmers who provided blood samples during both the non-exposure (baseline) and exposure periods. No correlation was found between the AChE and BChE activities (R^2^ = 0.25; *p* = 0.06). The majority of farmers (53.3%) had ±30% variation in AChE activity, 31.7% an increase in AChE activity greater than 30%, and 10 farmers had AChE depletion greater than 30%. One farmer had a 60% depletion in BChE during the exposure period (and a 29.2% AChE depletion), and two farmers had a BChE activity increase of over 50%; one with a 23% depletion in AChE, and the other with about the same AChE increase (Figure 3). For 48.3% of farmers, the depletion of one enzyme was followed by an increase in the other but, in most cases (51.7%), this variation was within ±25% for each enzyme. No significant correlation was found between enzyme alteration (depletion or overproduction) and work regimen, consumption of alcoholic beverages, hours of work, years of pesticide use, backpack application, reporting of adverse symptoms, and the use of PPD. 

Of the 17 residents who provided blood samples during the non-exposure and exposure periods, six (32.3%) had AChE depletions greater than 30% during the exposure period. BChE inhibition was within the normal range (up to 50%) in all cases (data not shown). All six residents with AChE depletion lived with the farmer who also had AChE inhibition.

## 4. Discussion

All 112 farmers participating in this study were directly involved with pesticides, either during solution preparation and/or its application in the field. All except one were men, reflecting the profile for the Brazilian rural population, and similar to the results obtained in other studies conducted in the country [3,26,30]. A different profile was found in certain regions of China, where most pesticide applicators were women, being potentially more vulnerable to pesticides [31]. In general, the education level of the farmers surveyed in this study was low (most had incomplete primary education or less), similar to other Brazilian regions [3,26,32], Colombia [12], and China [31]. In Greece, at least half of the tobacco farmers who applied pesticides had concluded primary education [33], and in the United States 55.8% of pesticide applicators in New York State had at least 12 years of schooling [34].

In Brazil, the legal term to define pesticides is *agrotóxico *(agrotoxic), which was introduced in the country in 1989 with the intention of giving farmers a clear message of the potential hazards of these products to human health. Although 26% of the farmers in this study referred to pesticides with this legal term, most preferred to use the term “poison”, indicating that the message intended by legislation was conveyed to this population. 

Most of the farmers reported storing the pesticide products in a reserved place outside the house and turning over the empty containers to the National Empty Container Processing Institute. This program began in 2001, and the number of empty pesticide containers turned over has increased continuously ever since, reaching about 17 thousand tons in 2010 [35]. High participation rates in the program were also reported by Faria *et al*. [3] in the state of Rio Grande do Sul (RS). In the state of São Paulo, half of the flower greenhouse workers interviewed declared returning the containers to specific waste collection posts [14]. In Mato Grosso do Sul (MS), Bigatão [36] reported that 34.8% of the farmers burned or buried the pesticide containers, and Recena *et al*. [26] found that 54.4% of farmers stored the empty containers in their homes. However, in the areas surveyed by this study, empty containers were found spread around the planting areas of those reporting having turned over the empty pesticide containers to the government program, indicating that the best practices are not fully carried out by these farmers.

Over 80% of the farmers considered that working with pesticides could impair their health, a similar perception found in MS [26]. In our study, the great majority of the farmers observed the direction of wind and chose the time of application. These practices are important to minimize human exposure in tropical regions since higher atmospheric temperatures increase the volatility of the chemicals, and thus their availability for inhalation and dermal absorption [37]. 

Recena *et al*. [26] observed that the majority of the farmers in MS wore hats, but less than half wore boots, masks, gloves or impermeable clothing. The farmers participating in our study seemed to be more aware of the importance of PPD use, and only 7.1% had never used them, similar to what was found in RS, where over 90% of farmers always used at least one PPD item [3]. In a province in Thailand [22], 64.4% of farmers did not use any type of PPD during their work, similar to what was found in Ethiopia (76.3%) [13]. In Spain, 65% of the workers used no personal protection or used it defectively [38]. Soares *et al*. [39] estimated that an unprotected Brazilian worker has a 72% greater chance of being poisoned when compared to those who use all PPDs. In our study, many farmers complained that certain PPDs were uncomfortable when used in warm weather; similar to what was found in other studies conducted in other tropical areas [11,22,30]. Although most of the farmers in our study declared reading the product label, almost 40% did not read the warning precautions, which give information over the use of PPDs and warnings on pesticide hazard. Furthermore, about 40% considered that the information they received from different sources were not sufficient to protect their health.

In this study, over 20% of farmers reported adverse symptoms after using pesticides, similar to the findings of Faria *et al*. [3] in RS (19.4%; the majority using tractor applicators). This percentage is much lower than that found by Recena *et al*. [26] in MS (60%; mostly backpack sprayer use). We did find a correlation between the reporting of adverse symptoms and the application of pesticides with backpack sprayers, used by 40% of the farmers. This technology, generally used on small farms, may increase the risk of developing adverse effects, as the farmer is greatly exposed to the pesticide spray [9]. We did not find a significant correlation between the consumption of alcoholic beverages and the reporting of any adverse symptom, a correlation found by Faria *et al*. [3].

According to Brazilian legislation, an AChE depletion of at least 30% indicates excessive exposure, and may be associated with an adverse effect [40]. In this situation, the recommendation is the removal of the exposed individual from further contact with pesticides until levels return to normal [41]. In the present study, 10 farmers had AChE depletions of over 30% during the exposure period, most of which over 50%, indicating potential poisoning [19,42]. Although the mean farmer AChE activity was significantly lower than the control group (about 15% lower), no differences between the mean farmer AChE activities during the non-exposure and exposure periods were found. This lack of sensitivity, when a population baseline is used to detect inhibition, is partially due to the normal interindividual enzyme activity variation, which is about 15–25% for BChE and 10–18% for AChE, and the intraindividual variation over time, which ranges between 6 and 3–7%, respectively [42]. Hence, an individual ChE activity baseline is necessary [21,24,43,44]. 

Only one of the farmers had a depletion of BChE activity higher than 50%, which is the level of health concern according to Brazilian legislation. The second highest inhibition level was 34.4%, lower than the depletion level in the USA that indicates the need to remove the farmer from the work setting (40% or higher) [45]. The lower sensitivity of BChE inhibition as a biomarker of exposure to OPs and CAR can be explained by the higher turnover of this enzyme when compared to AChE, for which the recovery of the activity is limited by the production of new erythrocytes, which takes over 120 days [22]. Thus, AChE remains depressed for a longer period [43,44]. In this study, all 10 farmers with enzyme depletion rates higher the cut off levels were from Taquara. 

Peres *et al*. [46] observed higher BChE activities during the exposure period when compared with the baseline activity (up to 42% higher), calling this phenomenon a reboot effect. The authors did not observe the same effect for AChE. In our study, the reboot effect was found for both AChE and BChE. An increase in AChE levels in the blood samples collected a few days or weeks after exposure may reflect the ability of the body to adapt to increased concentrations of acetylcholine at the nervous terminal after exposure to AChE inhibitors. This phenomenon may partially explain why the severity of illness after repeated exposures is not always proportional to the degree of AChE inhibition [47]. Furthermore, the reboot effect may also hide unsafe exposure when the individual baseline level is measured following a period of high exposure. 

Statistical analysis could not detect the effect of using PPD on enzyme depletion, nor a correlation between this depletion and the reporting of adverse symptoms. A close investigation of the questionnaires showed that seven of the 10 farmers who had AChE depletion activities higher than the cut-off levels reported having never used or occasionally used impermeable clothing, gloves and/or masks. Only four of them reported having any adverse symptoms. 

The OPs and CARs have substantial differences in their ability to inhibit either AChE or BChE [24]. In our study, we were not able to correlate enzyme activity with specific compound use. During our visits to the farms, it was clear that most farmers used multiple OPs on the same crop, mainly chlorpyrifos, triazofós, fentoate and/or methamidophos. Methamidophos was the OP most used in Brazil in 2009 [4], and was recently prohibited in the country [17]. 

Family agriculture settings are characterized by small properties, labor provided by family members, and the use of low technology equipment to apply pesticides, such as backpack sprayers and open tractors. In this system, houses are generally located just a few meters from the field, increasing exposure to pesticides of residents not directly involved in agricultural activities. Indeed, this study indicated that the resident group could be at risk from secondary exposure to pesticides, which can occur due to pesticide drift from the field, dust carried in from the farms (on clothing, skin or hair) and/or during the washing of farmers’ impermeable clothing. Women of child-bearing age are especially vulnerable to adverse effects of pesticides. According to the WHO [41], some pesticides may affect puberty hormones and gene expression, which can influence susceptibility to OPs. Eskenazi *et al*. [48] showed that shortened gestational periods were related to increased exposure levels to OPs in the latter stage of pregnancy in a Californian agricultural population. OPs have been shown to cross the placental barrier of laboratory rats and are eliminated through milk, exposing pups at an early developmental stage [49]. 

One main limitation to this work was related to the convenience sampling technique applied to recruit participants, which may lead to bias and may not reflect the entire population of the study area [50]. One source of bias could be that only farmers who were comfortable with their agricultural practices agreed to participate in the study. On the other hand, their concern about their agriculture practices and health might also have led some farms to join the study. Another limitation is the small number of participants. If we were to statistically define the number of participants, 285 farms from Taquara would need to be included in the study (5% sampling error at a 95% confidence level). During the systematic recruitment process, it became clear that this number would be impossible to reach within a reasonable time frame, mainly due to the difficulty of gaining access to a large number of farmers during a single visit, to the fact that the farmers are constantly changing activities and moving to other areas in the region, and that some were not using OPs and CARs for pest management. We faced a major difficulty in convincing managers in the Goianápolis area to participate in the study. This population received no technical support from the government and the managers of the planting areas probably did not feel at ease to participate in a study that could reveal agricultural practices that are not recommended. Although it was explained that the study had no legal intention, they were probably afraid to be held responsible for any problem we could have found among the farmers under their responsibility. The two managers who accepted were the first to be contacted, and it is likely that the farmers from these areas made comments in this regard to others farmers in the area, thus influencing their final decision. Many of them told the researchers that they had never taken a blood sample in their lives and were clearly afraid of the procedure during the study. Farmers from Taquara receive technical support from EMATER, and the institution’s outreach to farmers proved crucial to the recruitment process. We did not find, however, significant differences in the KAP study between the farmers from the two locations. All farmers with enzyme depletion rates higher than the cut-off levels were from Taquara, but it is possible that the number of farmers from Goianápolis was too small to detect relevant enzyme depletion.

Some studies have suggested that a true unexposed enzyme baseline measurement is reached around 30–60 days after exposure to anticholinesterase pesticides [24,43,45]. However, this period is difficult to be reached in locations with ongoing work, such as the areas investigated in this study, where the farmers apply pesticides during the entire year. We were able to establish a 15-day non-exposure period, during which the baseline could not have been reached, mainly for AChE, an enzyme with a lower turnover when compared with BChE [44]. Hence, it is possible that in some cases our sampling protocol did not allow the detection of enzyme depletion after exposure. 

## 5. Conclusions

The KAP study has shown that although most farmers were aware that pesticides can harm their health, many still use PPDs in an inappropriate manner, or not at all, during pesticide handling. Reasons for this may include the low levels of education of farmers, the fact that the use of complete PPDs is uncomfortable in hot weather, and that risk communication by government authorities and technical advisors did not take place or was ineffective in the region. Inhibition of ChE activity during the exposure period for some farmers was higher that what is considered safe, indicating that they might be at risk from exposure to pesticides during their agricultural activities. Furthermore, residents in family farming settings also showed AChE activity depletion and thus may also be at risk from secondary exposure to pesticides. It is necessary to increase awareness among the rural population in these areas of the risks from pesticide exposure through continuous local government education programs focused on the rational and safe use of pesticides, and the implementation of risk communication strategies to protect this population. 
